# An Analysis of the Multifaceted Roles of Heme in the Pathogenesis of Cancer and Related Diseases

**DOI:** 10.3390/cancers13164142

**Published:** 2021-08-17

**Authors:** Tianyuan Wang, Adnin Ashrafi, Pouya Modareszadeh, Alexander R. Deese, Maria Del Carmen Chacon Castro, Parinaz Sadat Alemi, Li Zhang

**Affiliations:** Department of Biological Sciences, The University of Texas at Dallas, Richardson, TX 75080, USA; txw130830@utdallas.edu (T.W.); axa131530@utdallas.edu (A.A.); pouya.modareszadeh@utdallas.edu (P.M.); alexander.deese@utdallas.edu (A.R.D.); mariadelcarmen.chaconcastro@utdallas.edu (M.D.C.C.C.); parinazsadat.alemi@utdallas.edu (P.S.A.)

**Keywords:** heme regulation, heme oxygenase, hemoproteins, cancer, diseases, drug targets

## Abstract

**Simple Summary:**

Heme is an iron-containing porphyrin that functions as a prosthetic group in hemoproteins and is involved in many biological processes. This review article summarizes (1) the varied effects of heme and heme oxygenase in tumorigenesis of different cancer types; (2) the molecular mechanisms of interaction of heme with regulatory and signaling proteins implicated in tumorigenesis, such as BACH1, PGRMC1, P53, CBS, sGC, and NOS; (3) the roles of altered heme levels and metabolism in the pathogenesis of diseases, including diabetes mellitus and Alzheimer’s dementia. Understanding the effects of heme in diverse cellular processes and disease progression identifies potential therapeutic targets and provides insights for developing novel treatment strategies.

**Abstract:**

Heme is an essential prosthetic group in proteins and enzymes involved in oxygen utilization and metabolism. Heme also plays versatile and fascinating roles in regulating fundamental biological processes, ranging from aerobic respiration to drug metabolism. Increasing experimental and epidemiological data have shown that altered heme homeostasis accelerates the development and progression of common diseases, including various cancers, diabetes, vascular diseases, and Alzheimer’s disease. The effects of heme on the pathogenesis of these diseases may be mediated via its action on various cellular signaling and regulatory proteins, as well as its function in cellular bioenergetics, specifically, oxidative phosphorylation (OXPHOS). Elevated heme levels in cancer cells intensify OXPHOS, leading to higher ATP generation and fueling tumorigenic functions. In contrast, lowered heme levels in neurons may reduce OXPHOS, leading to defects in bioenergetics and causing neurological deficits. Further, heme has been shown to modulate the activities of diverse cellular proteins influencing disease pathogenesis. These include BTB and CNC homology 1 (BACH1), tumor suppressor P53 protein, progesterone receptor membrane component 1 protein (PGRMC1), cystathionine-β-synthase (CBS), soluble guanylate cyclase (sGC), and nitric oxide synthases (NOS). This review provides an in-depth analysis of heme function in influencing diverse molecular and cellular processes germane to disease pathogenesis and the modes by which heme modulates the activities of cellular proteins involved in the development of cancer and other common diseases.

## 1. Introduction

**Figure 1:**
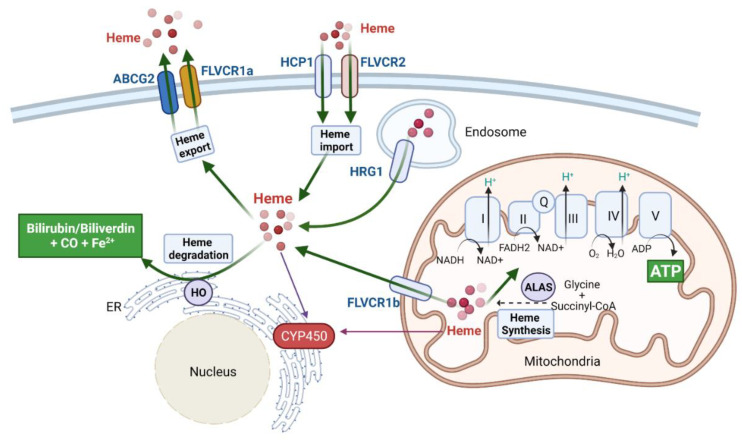
Heme metabolism in human cells. Human cells acquire heme in two ways. (1) Heme biosynthesis involves multiple enzymes that progressively convert glycine and succinyl-CoA to heme. (2) Heme uptake from the circulation to enterocytes is facilitated via heme importers HCP1/SLC46A1 and FLVCR2. Neuronal, hepatic, and red blood cells can directly take up heme from the bloodstream via HRG1. Heme serves as a prosthetic group in proteins involved in oxygen storage and usage, such as cytochrome P450. Heme degradation is carried out in the endoplasmic reticulum via HO. Heme exporters ABCG2 and FLVCR1a export heme out of cells to maintain cell heme level. (Created with BioRender.com).

Heme (iron–protoporphyrin IX) is a tetrapyrrole containing a central iron ion essential for living organisms ranging from bacteria to humans [1,2]. Heme serves as a prosthetic group of numerous hemoproteins, including hemoglobin (Hb), myoglobin, cytochromes, and enzymes (including most peroxidases and cytochromes P450s) that are involved in oxygen transfer, oxygen storage, electron transfer, drug metabolism, and oxidoreduction reaction catalyzation, respectively [3,4,5,6]. Heme also acts as a central signaling molecule that directly controls various proteins vitally involved in oxygen-related processes by regulating protein transcription, translation, assembly, and degradation [7,8,9,10,11]. Heme homeostasis is strictly regulated. Extracellular hemoglobin and labile heme are scavenged by Haptoglobin (Hp) and Hemopexin (Hx), respectively [12]. The intracellular heme levels are controlled via uptake, synthesis, export, and degradation (Figure 1). Hemoproteins can be taken up and denatured in endosomes, liberating heme that can be transported to the cytoplasm via heme responsive gene 1 (HRG1), whereas labile heme is taken up by heme carrier protein 1 (HCP1/SLC46A1) and feline leukemia virus subgroup C receptor (FLVCR) 2 [13]. Hopp et al. have summarized the commonly used methods for heme measurement, including spectroscopic methods, chromatography, and capillary electrophoresis, enzyme-/protein-based methods, and intracellular techniques [14]. However, the accurate measurement of heme derived from different sources (e.g., labile heme or hemoproteins), in varying concentrations and in complex composition of the different biological matrices (e.g., cellular milieu, blood, urine, or cerebrospinal fluid), is still an unsolved challenge. Advanced methods like MS-based or apo-HRP based assay can detect the nano- and picomolar levels of labile heme [14].

The heme biosynthetic pathway in humans requires eight enzymes that convert glycine and succinyl-CoA to heme. 5-aminolevulinic acid synthase (ALAS) is the rate-limiting enzyme of heme synthesis that initiates the pathway [1]. The export of heme is mediated by ATP binding cassette subfamily G member 2 (ABCG2) and FLVCR1a. Even though heme metabolism primarily happens in the cytosol and mitochondria, heme can be transported into the nucleus in association with carrier proteins such as biliverdin reductase (BVR) and regulate activities of nuclear proteins [15,16]. Heme oxygenase (HO) localizes at the smooth endoplasmic reticulum membrane (sER) and breaks down heme into biliverdin, carbon monoxide (CO), and ferrous iron (Fe^2+^) (Figure 1). After cleavage by certain proteases, the truncated HO-1 (*t*-HO-1) can translocate into the nucleus and function non-canonically [17]. Defects in heme metabolism and function are directly associated with porphyria, anemia, and neurological diseases. Heme metabolism, especially HO-1 expression, has been widely reported to be connected with cancers [18]. Notably, the ALAS1-mediated heme synthesis and FLVCR1a-mediated heme export are coupled and control the TCA cycle and OXPHOS [19]. Heme is an essential molecule that is involved in mitochondrial OXPHOS complexes formation [20]. Studies in the authors’ lab demonstrate that elevated OXPHOS activity in non-small cell lung cancer (NSCLC) cells is directly linked with mitochondrial heme levels [21]. Many other novel studies reveal the emerging role of heme, which directly functions as a regulator of cancers in various tissues and organs, as will be discussed in this review. Considering that heme is an essential and multifunctional molecule, heme may mediate the pathogenesis of diverse diseases via its action on various cellular signaling and regulatory proteins. In this review, we aim to provide a systematic overview of the prominent roles of heme in human cancer and cancer-related heme proteins, pathology, and diseases.

## 2. Heme and Cancer

### 2.1. Elevated Heme Levels Promote Lung Tumorigenesis

Growing evidence indicates that various cancers, including lung cancer, primarily rely on mitochondria to produce ATP, which fuels tumor proliferation, making mitochondrial respiration an attractive target in cancer therapy [22,23,24]. Lung cancer accounts for almost one-quarter of all cancer mortality in the United States, and NSCLC is the most common form of lung cancer, comprising about 80% of lung cancer cases [25]. Heme is a central molecule in mitochondrial metabolism, essential for OXPHOS Complex II, III, and IV function [23]. A series of studies in the authors’ laboratory demonstrate that elevated heme flux and function underlie NSCLC cells’ enhanced OXPHOS and tumorigenicity [21,26]. H1299, A549, H460, Calu-3, and H1395 NSCLC cell lines exhibit elevated heme synthesis and uptake as compared to two cell lines representing normal lung epithelial cells (HBEC30KT and NL20). These augmentations correlate with the increase of heme biosynthesis enzyme ALAS1, heme uptake protein HCP1/SLC46A1, and HO-1, respectively (Figure 1). Furthermore, the elevated heme metabolism in NSCLC cells leads to increased levels of OXPHOS complex subunits cytochrome c (CYCS) and cytochrome c oxidase subunit 4 isoform 1 (COX4I1). Elevated levels of heme and hemoproteins correlate with the increased oxygen consumption rates (OCR) and ATP generation [21]. The increased heme synthesis and uptake in NSCLC cells significantly elevate mitochondrial heme levels, but not the heme levels in other organelles [21]. Altogether, the enhanced mitochondrial heme levels and OXPHOS intensified oxygen consumption, ATP generation, and tumorigenic capabilities in NSCLC cells [21,27]. The essential roles of heme metabolism for NSCLC tumorigenic functions are verified by using heme-targeting agents heme-sequestering peptides (HSPs) [21,26] and cyclopamine tartrate (CycT) to inhibit heme flux and function [26,28]. By using the oxygen-enhanced multispectral optoacoustic tomography (OE-MSOT), which is an emerging noninvasive imaging modality that can monitor the tumor microenvironment, the authors’ lab demonstrates that HSPs and CycT efficiently normalize the tumor microenvironment, including angiogenic function, tumor vasculature, tumor oxygenation, and ATP generation, which ultimately leads to the suppressed proliferation and metastasis of NSCLC cells [26]. The studies of the increased need for heme in NSCLCs and heme-targeting drugs extend a new potential strategy for lung cancer treatment. Overall, these studies highlight the importance of heme in lung cancer and heme scavenging as a potential therapeutic approach.

### 2.2. Heme Synthesis, Export, and Catabolism, along with Dietary Heme Intake, Play a Role in Pancreatic and Colorectal Cancer

Changes in heme metabolic pathways resulting from alterations in levels of heme synthesis and catabolism have been observed in pancreatic cancer. Two recent studies using CRISPR genetic screens examined the expression of heme synthesis genes in pancreatic cancer [29,30]. Zhu et al.’s study shows that pancreatic cancer proliferation in vivo is highly dependent on heme synthesis, while HO-1 is also substantially upregulated in tumors and hypoxic cultured pancreatic cancer cells [29]. Knockout of *Hmox1*, the gene that encodes HO-1, also partially rescues proliferation of pancreatic cancer cells deficient in hydroxymethylbilane synthase (*Hmbs*), implying that the dependency on heme synthesis may be partially caused by environmentally-induced upregulation of HO-1, resulting in increased heme degradation [29]. In addition, targeting heme synthesis inhibits the growth of pancreatic cancer xenografts [29]. Pancreatic ductal adenocarcinoma cells in vivo showed metabolic dependencies on multiple enzymes of the heme biosynthesis pathway in a mixed population with heme biosynthesis deficient and wild type cells [30].

Colorectal cancer (CRC) has been reported to exhibit altered heme synthesis, export, and catabolism, and is potentially related to dietary heme intake. Heme exporter FLVCR1 is overexpressed in humans and mice CRC cells, and FLVCR1a-silenced cells show slower proliferation [19]. In addition, the FLVCR1a-silenced cells also showed decreased levels of ALAS1 compared to controls, while overexpression of FLVCR1 resulted in increased ALAS1 expression, suggesting that elevated heme export plays a role in the maintenance of heme synthesis in these cells, likely resulting in downregulation of the TCA cycle as the TCA intermediate succinyl-CoA is consumed for heme synthesis [19]. FLVCR1 silenced CRC cells show increased OXPHOS and TCA cycle flux compared to controls [19]. Thus, the elevated activities of the heme synthesis-export system in CRC downregulates the TCA cycle and oxidative metabolism and promotes tumor growth [19]. Many studies of CRC have also examined the role of dietary heme iron in CRC carcinogenesis. In 2018, International Agency for Research on Cancer (IARC), the cancer agency of the World Health Organization, released a monograph that concluded that, based on limited dietary studies and mechanistic evidence of carcinogenicity, red meat is probably carcinogenic to humans, with the strongest association seen in CRC [31]. Various mechanisms have been proposed to explain this association, including effects caused by interactions between heme iron and colonic epithelial cells, the action of heterocyclic amines (HCAs), and polycyclic aromatic hydrocarbons (PAH) formed by pyrolysis during meat smoking [32,33,34], or an inflammatory response to the incorporation of N-Glycolylneuraminic acid (Neu5Gc) into cell surface glycoconjugates of healthy epithelial cells by circulating anti-Neu5Gc antibodies [35,36,37].

Interaction of heme with fatty acids results in lipid peroxidation and production of aldehydes, such as 4-hydroxynonenal (4-HNE) and malondialdehyde (MDA) [38]. The levels of 4-HNE are highly elevated in the colon, urine, and fecal water of rats fed a diet of heme and safflower oil [38]. Adenomatous polyposis coli (*APC*) gene is an early and frequently mutated gene in colorectal carcinogenesis. The formation of 4-HNE or MDA via lipid peroxidation significantly induces *APC* gene mutation as exposure to these molecules triggers apoptotic effects in healthy cells but not *APC* mutant cells, leading to development of CRC [39,40,41,42]. A recent study has revealed that dietary heme iron, but not N-nitroso compounds (NOCs) or HCAs, significantly induces precancerous lesions in carcinogen-induced rats and *APC* mutant mice [41]. When imported to the nucleus, the transcription factor nuclear factor erythroid 2-related factor 2 (Nrf2) forms a heterodimer with musculoaponeurotic fibrosarcoma (MAF) proteins which bind to promoters with antioxidant response elements to initiate transcription of many antioxidant genes, including HO-1. *APC* mutant cells show increased nuclear localization of Nrf2 basally and in response to 4-HNE exposure compared to normal cells, creating a selective carcinogenic effect in which *APC* mutant cells protect against 4-HNE exposure by inducing the production of antioxidant compounds through the Nrf2/Keap1/ARE signaling pathway [41]. Further studies are needed to fully explore the degree to which heme and other potential mechanisms contribute to the observed association between red meat and colorectal carcinogenesis, including the degree to which byproducts of lipid peroxidation such as 4-HNE may cause a selective effect that induces carcinogenesis.

Overall, these studies demonstrate that heme synthesis, export, and catabolism may be altered in pancreatic cancer and CRC, affecting cellular metabolic pathways, and that dietary heme may play an additional role in colorectal carcinogenesis.

### 2.3. Paradoxical Roles of HO-1 in Cancer

The inducible heme oxygenase HO-1 is a key enzyme that is involved in heme degradation. It responds to electrophilic stimuli, including oxidative stress, cellular injury, and diverse diseases [43]. HO-1 induction under various pathological stresses impacts carcinogenesis and tumor progression through multiple pathways that involve heme, biliverdin, CO, and Fe^2+^ [43,44]. The functions of HO-1 in cancer are paradoxical and are highly dependent on tumor microenvironment and tumor type [44].

The pro-tumorigenic role of HO-1 has been reported in lung cancer, gliomas, gastrointestinal cancers, thyroid cancer, genitourinary cancers, melanoma, and hematological malignancies [17,45]. These effects of HO-1 in cancer, which are carried out via *HMOX1* regulation, modulating tumor microenvironment, and translocating to the nucleus, have been thoroughly reviewed [17,44,46]. HO-1 and heme degradation product CO have been shown to induce angiogenesis in tumors, possibly through stimulation of vascular endothelial growth factor (VEGF) expression [47,48,49,50,51]. The pro-angiogenic protein VEGF has a notable correlation with Nrf2 and HO-1 in patients with gastric cancer. Therefore, targeting VEGF by the Nrf2/HO-1 signaling pathway can positively regulate the angiogenesis in GC [47,52]. The mechanism of VEGF stimulation involving CO has been proposed to be mediated by hypoxia-inducible factor 1-alpha (HIF-1α) [53,54,55]. The activity of HO-1 may also allow cancer cells to avoid immune response via its expression both in cancer cells and in other cells in the tumor microenvironment including dendritic cells, as well as tumor associated macrophage (TAM) cells that can prevent activation of cytotoxic T cells [46,56]. HO-1 inhibits the maturation of dendritic cells, protecting tumors from T cell-based immune response [57,58,59,60]. In MIA PaCa-2 and PANC-1 pancreatic cancer cell lines, overexpression and inhibition of HO-1 correlated with a corresponding increase or decrease in cell proliferation and sonic hedgehog (SHH) signaling [61]. Heme degradation via HO-1 expression has been associated with decreased overall survival rate and relapse free survival in patients with pancreatic cancer [62]. The HO system also plays a critical role in chemoresistance and development of brain cancer, as evidence points towards heme as important for the maintenance of the peripheral nervous system that innervates tumors [63]. Studies suggest that metabolism of some neuropeptides and neurotransmitters is regulated by HO-1 and heme, which is crucial for nerve-cancer cell cross-talk. This contributes to the tumor microenvironment and promotes cancer progression [64]. Expression of HO-1 in tumors of the nervous system is reported to correspond with the aggressive nature of cancer [65,66]. A recent publication by Consonni et al. has discussed a new role of HO-1 in response to immunological stress, such as during tumor progression. They have discussed how bone marrow expresses a unique kind of HO-1 which is expressed in monocyte or macrophages. This population, activated by Nrf2, localizes in tumor lesions, a signal that is coordinated by p50 NF-κB–CSF-R1–C3aR axis. This promotes more HO-1 expression, immunosuppression, and therefore lower survival rate of melanoma patients [67].

Conversely, in some tumors, HO-1 may inhibit tumorigenesis. Prostate cancer, NSCLC, hepatocellular carcinoma, breast cancer, pancreatic cancer, and CRC have been shown in studies to be inhibited by HO-1 expression [45,68,69,70,71,72,73,74,75]. This tumor-suppressive role has been suggested to be due to different pathways, including matrix metallopeptidase 9 (MMP-9) and matrix metallopeptidase 13 (MMP-13) down-regulation in lung mucoepidermoid carcinoma [70], as well as VEGF and MMP-9 downregulation in pancreatic and prostate cancers [73,74,75]. Natural compounds that may synergize with conventional cancer therapies, such as Sageretia thea extracts, Ginnalin A (red maple), and fisetin (strawberries), have been shown to decrease cancer cell viability and inhibit colony formation and cell migration in CRC and metastatic breast cancer due to mechanisms of HO-1 upregulation via Nrf2 [45,76,77,78].

Furthermore, HO-1 mediates ferroptosis in hepatic cancer, head and neck cancer, astrocytoma, oligodendroglioma, glioblastoma multiforme, and breast cancer through iron accumulation and reactive oxygen species (ROS) generation [79,80]. Depending on the degree of ROS production, contradictory roles of HO-1 in cancer may be observed [43]. While moderate upregulation of HO-1 expression can be beneficial due to the antioxidant properties of HO-1 activity, higher levels of expression may induce ferroptosis in cancer cells due to the accumulation of reactive iron [81,82,83,84]. In breast cancer, HO-1 displays anti-tumor activities, including reducing tumor size and prolonging the overall survival time of patients [85]. The activation of HO-1 involves the epithelial-mesenchymal transition and induces the apoptosis and cell cycle arrest of breast cancer cells. Contrarily, the *t*-HO-1 that localizes in the nucleus promotes breast cancer growth and invasion independent of HO-1 activity [85]. The localization of HO-1 is likely to be associated with the dual role of HO-1 in cancer [17].

Overall, more in-depth investigations are still required to further understand the multifaceted role of HO-1 in cancer. HO-1 expression likely differs in various cancer types; thus, modulation of HO-1 expression may be useful in anti-cancer treatments. The HO-1/heme axis is likely to be a promising clinical tool in cancer therapeutics.

### 2.4. Heme Acts as a Regulator of Circadian Rhythm Implicated in Cancer

The body has a natural, repetitive sleep wake cycle known as the Circadian Rhythm (CR), which regulates many metabolic and cellular processes. Cancers and other diseases, including metabolic dysregulation, have been shown to be related to altered CR [86,87,88,89,90,91]. In fact, several epidemiological studies have linked circadian disruption, for example, in the case of shift work, to increased occurrence of cancers [92,93,94,95,96,97,98,99,100,101], and the IARC lists night-shift work, which disrupts CR as a carcinogen [97,102]. The Circadian Clock is regulated via circadian locomotor output cycles kaput (CLOCK) and Brain and muscle ARNT-Like 1 (BMAL 1), which are basic helix-loop-helix PER-ARNT-SIM transcription factors [103]. Nearly all tumor types exhibit alteration in the expression of *CLOCK* genes [104]. Heme binding to CLOCK has been shown to disrupt CLOCK binding to E-box DNA target [103]. Furthermore, heme binds to Period circadian protein homolog 2 (PER2), which regulates CR in the suprachiasmatic nucleus, and mediates the stability of PER2 [91,105,106,107]. Heme is also a regulator of CR through interactions with nuclear receptor subfamily 1 group D member 1 (Rev-Erbα), which is a heme receptor that coordinates CR and metabolism [108]. Unorphaned nuclear receptor Rev-Erbα, found in 2007 to bind heme, is a member of the nuclear hormone receptor (NHR) superfamily implicated in CR and metabolism [108,109,110]. Rev-Erbs, sharing a high degree of homology and redundancy, are unique NHRs that act as transcriptional repressors via corepressor recruitment of nuclear receptor corepressor 1 (NCoR1) [108]. The Kd of Rev-Erb affinity for Fe^3+^ heme was determined to be between 0.35 and 3.52 μM using isothermal titration calorimetry with a roughly 1:1 heme to receptor binding ratio, bound to axial histidine and heme regulatory motif (HRM) associated cysteine ligands [109,111,112]. Heme is necessary for the complexing of Rev-Erbα and NCoR1, by binding to Rev-Erbα and supporting co-repressor recruitment [106,109,110,113]. Heme metabolism is also closely related to CR. ALAS1, the key heme synthesis enzyme, is CR regulated by BMAL1 and NPAS2 (a CLOCK paralogue) [113]. Heme degradation by HO-1 and 2 is also regulated by the circadian clock, and inhibition of heme degradation alters CR [113]. CO, a main heme degradation product, has also been shown to modulate transcription by suppressing target gene binding of CLOCK-BMAL1 [113].

Altering iron content in mouse diet affects CR and gluconeogenesis by modifying heme levels, affecting Rev-Erbα and NCoR1 complex formation [91]. CR dysregulation is implicated in the occurrence and metastasis of NSCLC by reducing circadian controlled hepatic leukemia factor (HLF), which is dramatically reduced in early relapsed NSCLC and upregulation of which inhibits lung colonization and metastasis [114]. Resistance to Bevacizumab, a major VEGFA antagonist used in cancer therapy to inhibit angiogenesis, has been shown to be related to Rev-Erbα binding to retinoic acid receptor-related orphan receptor alpha (RORA) responsive element adjacent to circadian clock key regulator BMAL1 E-box in VEGFA proximal promotors, which increases VEGFA mRNA and protein expression [115]. High BMAL1 protein expression is clinically correlated with non-response to Bevacizumab combination therapy and reduced progression-free survival, with SNPs in *BMAL1* gene correlated with shorter survival in combination therapy patients [115]. Rev-Erbα siRNA is shown to decrease VEGFA synthesis, pointing towards Rev-Erbα and BMAL1 as targets to prevent anti-angiogenic therapy resistance [115].

### 2.5. Heme Controls the Activities of a Variety of Key Regulators Underlying Diverse Cancers

The chemical complexity of heme iron ion and porphyrin performs critical regulatory roles in many vital human proteins. The iron ion in heme can be coordinated by axial ligands such as amino acid residues cysteine, histidine, methionine, and tyrosine [116,117]. Heme controls most of the key regulators through HRMs [118]. Each HRM contains one cysteine–proline (CP) motif, in which the cysteine residue coordinates the iron ion in heme and is crucial for heme binding [9,119]. Notably, non-CP motif heme-coordinating residues can also serve as heme-binding sites in the heme regulatory events.

**Figure 2:**
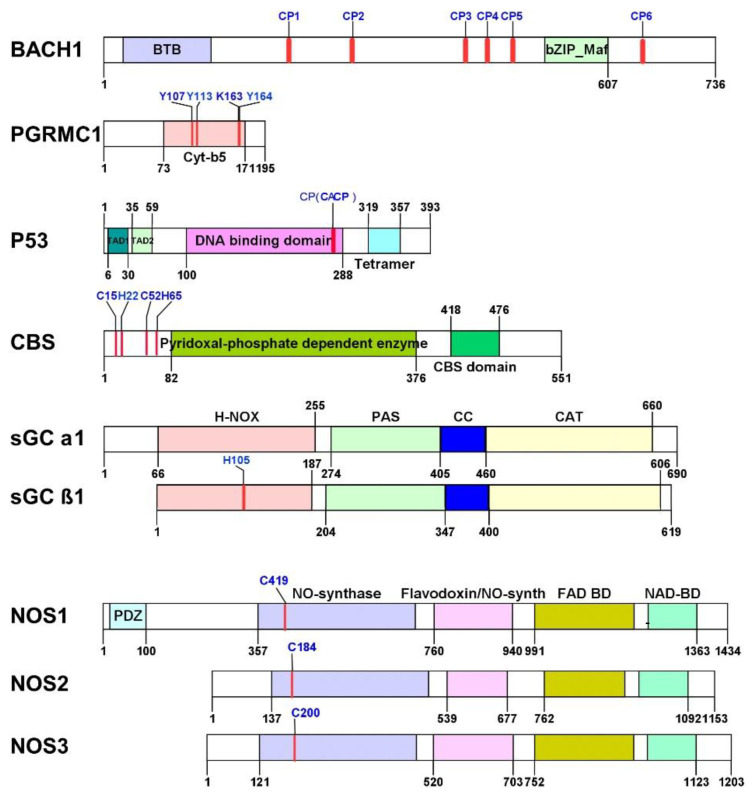
The domain structure of heme regulated signal transducers and regulators. Red lines represent the location of heme-binding sites. BACH1: BTB domain (Broad-Complex, Tramtrack and Bric a brac), bZIP_Maf domain (Basic leucine zipper domain, Maf-type). PGRMC1: Cyt-b5 (Cytochrome b5-like) heme/steroid binding domain. P53: TAD (Transcriptional Activation Domain) 1 and 2, DNA binding domain, Tetramer (Tetramerization domain). CBS: Pyridoxal-phosphate dependent enzyme, CBS domain. sGC α1 and β1 subunit: that each contain four domains: H-NOX (heme nitric oxide and oxygen-binding domain), PAS (Per-Arnt-Sim domain), CC (coiled-coil domain), and the CAT (catalytic domain). NOS: NO-synthase, Flavodoxin/NO-synthase, FAD binding domain, and NAD binding domain.

Heme regulates diverse cellular processes through directly binding and mediating key regulators, including heme-responsive transcription regulators BACH1 [120] and BACH2 [121]; tRNA synthetases tryptophan-tRNA ligase (TrpRS) [122] and arginine-tRNA ligase (ArgRS) [123]; microRNA processing protein DiGeorge syndrome critical region 8 (DGCR8) [124]; electron transfers six-transmembrane epithelial antigen of prostate (STEAP) 1 [125], STEAP3 [126], and cytochrome b reductase 1 (Dcytb) [127]; iron regulatory protein (IRP) 1 and IRP2 [128]; heme metabolism-related proteins ALAS [129] and HO-2 [130]; circadian-rhythm related proteins Rev-erbα and Rev-erbβ [109], neuronal PAS domain-containing protein 2 (NPAS2) [131], CLOCK [103], PER2 [132], and Cryptochrome-1 (CRY1) [133]; Alzheimer’s disease-related Aβ peptide [134]; immune and inflammatory responses regulator IL-36α [135]; potential cancer therapeutic target proteins BACH1 [136,137], PGRMC1 [138], P53 [139], CBS [140], sGC [141], and NOS [142]. This section summarizes the novel regulatory roles of heme in multifunctional signal transducers and regulators involved in various cancer diagnoses and treatments (Figure 2 and Table 1).

#### 2.5.1. BACH1

BTB and CNC homology 1 (BACH1) protein is a heme-dependent transcription factor involved in oxidative stress response, heme homeostasis, the cell cycle, senescence, mitosis, and angiogenesis [156]. BACH1 forms heterodimers with a small Maf protein that binds to the Maf recognition elements (MAREs) and represses its target genes. BACH1 contains [157] six HRMs [158]. Heme binds to BACH1 via HRMs and regulates BACH1 activity in three ways. (1) Heme binds to HRMs 3-6, causing dissociation of BACH1 from MAREs [120]. (2) Heme induces the nuclear export of BACH1 and requires HRM3 and HRM4 [159]. (3) Heme promotes the HOIL-1 and Fbxo22-mediated ubiquitination and degradation of BACH1 via HRMs 3-6 [11,160]. BACH1 senses increased heme levels in cells and dissociates from MAREs, making MAREs available for the activator factor Nrf2 and activating HO-1 [120]. This mechanism generates a feedback loop whereby heme affects BACH1/Nrf2 antagonism [161]. Two recent studies illustrated the importance of BACH1 stabilization in lung cancer metastasis. The mutation of Keap1, an Nrf2 negative regulator, activates Nrf2 and promotes the expression of HO-1. The elevated HO-1 inhibits the heme-and Fbxo22 (a heme-regulated ubiquitin ligase)-mediated degradation of BACH1 and further promotes lung cancer metastasis [160]. Furthermore, Weil et al. suggest that treatment with the antioxidants N-acetylcysteine and vitamin E leads to decreased levels of heme, which stabilizes the transcription of BACH1 and promotes glycolysis-induced metastasis in KRAS-driven lung cancer [136]. Their studies provide new therapeutic approaches for lung cancer related to heme metabolism. Interestingly, while mitochondrial heme promotes lung cancer proliferation and metastasis through elevating OXPHOS and promoting angiogenesis, HO-1 regulated heme seems to play a positive role in the inhibition of lung cancer metastasis through inhibiting BACH1. The opposite roles of heme in lung cancer indicate that heme may coordinate lung cancer progression in distinct regulating mechanisms. The study by Lee et al. shows that combination therapy targeting BACH1 and mitochondrial metabolism suppresses breast tumor growth [137], which further illustrates the central regulatory role of heme in different types of tumors. More studies are required to understand the complex mechanisms underlying the regulation of tumor growth by heme and heme metabolism.

#### 2.5.2. PGRMC1

PGRMC1 belongs to the membrane-associated progesterone receptor (MAPR) family and may function in the endoplasmic reticulum and mitochondria [162,163]. A significant amount of studies have reported that PGRMC1 is highly expressed in cancers including renal cell cancer [164,165], colon cancer [138], lung cancer [166], ovarian cancer [167], cervical cancer [168], breast cancer [169,170,171], and head and neck cancer [172]. Compelling evidence implies that PGRMC1 represents a promising target for cancer therapy.

Site-directed mutation experiments and crystallographic analyses identify four heme-binding residues—Tyr113, Tyr107, Lys163, and Tyr164—in PGRMC1 [138,146,147]. The Tyr113 residue plays a crucial role in a unique heme-dependent dimerization of PGRMC1 (binding Kd = 50 nM) [138]. The five-coordinated heme iron by Tyr 113 has an open surface which allows PGRMC1 to form a stable dimer through hydrophobic heme–heme stacking (dimerization Kd << 3.5 uM) [138]. PGRMC1 may act as heme transporter because Y113-heme bound structure is similar to heme bound structure of bacterial heme transport proteins such as HasA (heme acquisition system protein), ShuT (periplasmic heme transport protein identified from *Shigella dysenteriae*), and HTP/PhuT (heme transport protein from *Pseudomonas aeruginosa*) [173]. The heme-mediated dimerization is essential for PGRMC1 to bind and regulate epidermal growth factor receptor (EGFR) and cytochromes P450. Kabe et al.’s study reveals the importance of heme in regulating EGFR and cytochromes P450 mediated tumorigenesis of colon cancer cells and hepatoma cells [138]. Furthermore, they show that PGRMC1 dimerization is related to HCT116 cell chemoresistance to anti-cancer reagents erlotinib and doxorubicin [138]. The phosphorylation of heme chelation site Tyr 113 may contribute to the regulation of heme-mediated PGRMC1 dimerization [174]. PGRMC1 is recently identified as a protein partner of ferrochelatase (FECH) [175]. FECH catalyzes the terminal step of heme biosynthesis in mitochondria, and it is also involved in cellular iron metabolism [173]. Piel et al. proposes that PGRMC1 regulates the release of heme from FECH by stabilizing the “product release” conformational state of FECH [175]. Altogether, these recent studies reveal a possible heme–PGRMC1 regulatory loop where heme biosynthesis and heme-mediated PGRMC1 dimerization are closely connected and tightly regulated, implicating heme’s role as a critical effector molecule in the signal network surrounding PGRMC1 [138,173,174,175]. Characterization of the heme–PGRMC1 regulatory pathway could potentially lead to novel therapeutic approaches for cancer and other diseases involving PGRMC1.

#### 2.5.3. P53

Known as “the guardian of the genome”, the transcriptional factor P53 has been extensively studied due to its vital role in maintaining genomic stability and preventing genome mutation [176]. P53 suppresses tumorigenesis and regulates tumor chemoresistance through transcriptional regulation of diverse genes that encode for proteins, such as cyclin-dependent kinase inhibitor p21, which is essential for G1 arrest induced by DNA damage [177], apoptosis regulator Bax [178], and hypoxia central regulator HIF that is involved in tumor angiogenesis [179]. Many of the P53 regulated proteins are involved in different steps of the cancer process. Inactivation of P53 via mutation and alternative mechanisms occurs in almost 50% of all human tumors [179,180]. Heme directly interacts with P53 through Cys275 and Cys277, which are located at the C-terminal HRM in P53 (Kd ~1.2 μM) [139]. Shen et al.’s study suggests that heme binds to P53 and mediates P53 destabilization through two mechanisms: (1) directly triggering nuclear P53 degradation through the Ub-proteasome system. (2) unmasking P53 C-terminal nuclear export signals (NESs) and inducing the nuclear export of P53, which leads to the cytosolic degradation of P53. Deprivation of iron suppresses human colon carcinoma development in a P53-dependent manner [139]. Their study indicates that heme–P53 seems to underlie the molecular mechanisms involved in iron deprivation-based chemotherapy. Furthermore, the *P53* knockout embryonic stem cells (ESCs) exhibited elevated HO-1 protein levels compared to the WT cell line. Thus, P53 is likely to modulate HO-1 stability in ESCs, and the interplay between P53 and HO-1 may be involved in a complex mechanism of ROS balance regulation [181]. As HO-1 is primarily involved in the oxidative degradation of cellular heme, these studies indicate a P53-heme regulation loop may exist.

The P53 activity is also closely related to other heme-regulated key factors. A recent study shows that PGRMC1 knockdown promotes the stabilization of P53 protein in human pluripotent stem cells (hPSCs). The reason may be that the PGRMC1 knockdown abolishes heme transferring between sub-organelles [182]. BACH1 interacts with P53 on chromatin, inhibits the transcription of a subset of P53 target genes, and further inhibits the senescence in response to cellular oxidative stress [183]. The BACH1-P53 interaction is proposed to be regulated by a tumor suppressor p19^ARF^ [184]. These studies provide a possibility of heme being functional in a regulatory network involving P53 in cancer cells and mediating tumor growth and progression. This makes heme a promising target in tumor treatment.

#### 2.5.4. CBS

CBS is a pyridoxal 5′-phosphate (PLP) enzyme that catalyzes the transsulfuration pathway to convert homocysteine (Hcy) and serine into cystathionine and regulates hydrogen sulfide (H2S) metabolism [185,186,187]. It is a heme protein that conducts critical cellular bioenergetic-related processes, such as redox regulation [188] and mitochondrial homeostasis [189]. Defects in CBS expression lead to alternating Hcy levels and cause diseases such as hyperhomocysteinemia. On the other hand, increased activity of CBS in Down syndrome patients significantly decreases the availability of Hcy and increases levels of the cellular H_2_S [190,191,192]. The higher H_2_S levels are induced by elevated CBS expression and are also associated with the proliferation of multiple tumor types such as colon [193,194,195], ovarian [196,197], breast [198], and prostate cancer [199]. Human CBS contains an N-terminal heme-binding domain followed by a catalytic core and a regulatory domain [200]. Residues Cys52 and His65 of CBS form a hydrophobic pocket and axially coordinate the heme iron [148,149]. Heme is not directly involved in CBS catalysis [201]. Recent studies propose two mechanisms of how heme regulates CBS activity. (1) When in a ferrous state, CBS heme binds to NO and CO, and functions as a redox sensor that impairs CBS activity [149,202,203]. (2) CBS heme in a ferric state interacts with mercury compounds, leading to heme release and loss of enzymatic activity [204]. The NMR study carried out by Kumar et al. identifies a second heme-binding site at Cys15 and His22 (Kd = 2.18 ± 0.64 μM) located in the N-terminal intrinsically disordered protein region (IDPR) of CBS (1-40 aa) [140]. Their UV/Vis study indicates that the CBS IDPR involves transient heme interactions and forms a hexacoordinated complex that may increase enzyme efficiency (~30%) [140]. The novel studies of CBS in different types of cancer models suggest CBS is a promising anti-tumor therapeutic target. However, as an essential enzyme involved in a central physiological process, CBS targeted inhibitors may accumulate excess metabolite (i.e., Hcy), leading to severe side effects. Targeting the heme-binding domain may inhibit CBS activity without abolishing the enzymatic activity of CBS, which could be a potential direction for CBS-targeting drug invention.

#### 2.5.5. NO Signaling Related Hemoproteins

sGC is a key enzyme of the nitric oxide (NO) signaling pathway that converts GTP to the second messenger cGMP and exerts effects in many downstream processes [1]. sGC is a heterodimer with an α and β subunit that each contain four domains: H-NOX, PAS, CC, and CAT (Figure 2) [150]. In mammals, there are four isoforms of the subunit: α1, α2, β1, and β2 [205]. Only the H-NOX of β subunits has the heme-binding capability, and it serves as a sensor of NO which binds to heme iron and triggers the structural rearrangement of the sensor module and causes a conformational change to initiate the first step in sGC activation [150,151,152]. sGC is a prototypical hemoprotein that plays numerous important regulatory roles in NO signaling-related physiological events, such as vascular smooth muscle relaxation, platelet aggregation, and neurotransmission [150].

Considering the importance of NO signaling in the cardiovascular system, sGC has been well studied as a therapeutic target in cardiovascular, cardiopulmonary, and cardiorenal diseases [206,207]. Recent studies show that sGC expression is deficient in human breast cancer cells [141,208] and overexpression of α1 and β1, the two most common sGC subunits, inhibited the growth of both MDA-MB-231 cells and MDA-MB-231 xenografts in nude mice [208]. The histone deacetylase 3 is likely to be an endogenous antagonist of sGCβ1 expression in breast cancer and other vascular-related diseases [141]. Mohammadoo-Khorasani et al. have reported that the variations in the expression of sGC subunits alternative splicing forms and isoforms levels may be potentially connected with the sGC anti-tumor activity in breast cancer [205,209]. The sGC stimulators significantly increase the sensitivity of head and neck squamous cell carcinoma (HNSCC) cells to the hemotherapeutic drug Cisplatin [210].

NOS are hemoproteins that catalyze the reaction producing NO from l-arginine. The nitric oxide synthase protein family includes neuronal NOS (NOS1/nNOS), inducible NOS (NOS2/iNOS), and endothelial NOS (NOS3/eNOS) [211,212]. NO is an important signaling molecule with roles in the signaling of angiogenesis, inflammation, and the cell cycle [142,213]. All three varieties of NOS are homodimers, and heme must be available for dimerization to occur [153,154,155,212,214]. The presence of nitric oxide has been shown to interfere with heme insertion into and dimerization of NOS [211,215,216,217], and may also inactivate FECH, the final enzyme in heme synthesis, by interaction with its iron–sulfur complex [218,219,220]. Heme can also be degraded by reactive oxygen species, inhibiting NOS dimerization [155]. As a result, heme plays a role in the regulation of NO signaling.

NOS2/iNOS has been found to be upregulated in many cancers, including gastric cancer [221], breast cancer [222], CRC [213,223,224], pancreatic cancer [225], and prostate cancer [226]. A recent review by Mishra et al. identified many additional studies demonstrating increased expression of NOS in cancers [227]. Despite this upregulation in some cancers, NOS proteins also have a role in apoptosis. When NO is released in an oxidative environment, it can interact with O_2_^−^ to form nitrogen dioxide (NO_2_) or peroxynitrite (ONOO^−^), resulting in cytotoxicity in exposed cells at high concentrations [228]. Additionally, exposure of cells to NO leads to phosphorylation of P53 resulting in apoptotic effects [229,230,231]. However, other studies have found that low concentrations of NO protect against apoptosis through S-nitrosylation of metalloproteins such as caspase-3, caspase-9, and c-Jun N-terminal kinase [232,233].

NO signaling has both tumorigenic and antitumorigenic effects in cancer. Heme possesses unique signaling and structural properties that potentially enable it to coordinate the NO signaling regulation in cancers.

### 2.6. Heme Promotes Angiogenesis Implicated in Tumorigenesis

Angiogenesis refers to the formation of new blood vessels from pre-existing vessels due to changes in growth of endothelial cells (ECs) and an imbalance between pro- and anti-angiogenic factors [234]. Angiogenesis is essential under physiological conditions for wound repair and endometrial hyperplasia during the menstrual cycle and development [234,235]. However, it also plays an important role under pathological conditions such as tumors, hepatitis, diabetes, etc. [234]. EC proliferation and apoptosis balance is necessary for the mediation of tumor angiogenesis. Moreover, angiogenesis is highly required for fast and invasive tumor growth and metastasis [235], which is characterized by the formation of serpentine, disorganized, friable, and extremely permeable blood vessels. Under hypoxic conditions and lack of nutrients, which boosts the expression of inflammatory signals and cytokines, vascular cells are recruited for the formation of tumor vasculature [236].

ECs accommodate distinctive cellular capacities required for angiogenesis like multiplication, movement, and vascular penetrability [237]. High levels of ROS inhibit ECs migration and lead to impaired angiogenesis. Recent studies have revealed the critical role of heme in angiogenesis. The heme dependent transcription factor BACH1 inhibits HO-1, enhances mitochondrial ROS production, and competitively inhibits β catenin, which leads to inhibition of VEGF expression and angiogenesis [238,239,240]. Moreover, moderate levels of heme can induce EC proliferation; however, elevated heme levels may inhibit it. Medium heme supplementation (20 μM) has the peak effect in hyperoxic and normoxic conditions and can inhibit BACH1 expression, promote VEGF expression, and relieve hyperoxia-induced inhibition of proliferation, migration, and angiogenesis in human microvascular endothelial cells (HMEC-1) [144]. Furthermore, genetic deletion of BACH1 promotes angiogenesis under increased oxidative stress conditions after hindlimb ischemia [145].

Additionally, FECH is a key enzyme for heme synthesis that inserts Fe^2+^ into protoporphyrin IX to supply protoheme IX in mitochondria [1,241]. The FECH loss changes the shape and mass of mitochondria and leads to notable oxidative stress. However, the addition of heme partly rescues phenotypes of the FECH barricade [241,242]. These results present a novel link between heme metabolism, mitochondrial function, and angiogenesis. Additionally, heme exporter protein FLVCR1a expression is essential for proper angiogenesis, and its loss in ECs increases levels of intracellular heme, promoting cell death by paraptosis and preventing the formation of the functional microvascular network, which leads to extensive hemorrhages and embryonic lethality in FLVCR1a null mouse embryo cells. Considering that elevated intracellular heme levels cause paraptosis, this mechanism can be exploited as a valuable alternative to reduce tumor growth and angiogenesis [243]. It is possible that drugs that target increased heme synthesis, block heme export, and/or block heme catabolism might have anti-angiogenic effects and can be used to kill apoptosis-resistant cells in cancer and other enhanced aberrant vascularization [243]. Heme-targeting drugs can act as a potential angiogenesis inhibitor in drug-resistant tumors, such as NSCLC [26]. Nonetheless, additional studies that target the heme metabolic machinery would be recommended for the development of potential therapeutic drugs against cardiovascular diseases and angiogenesis in tumors.

Notably, many studies have linked elevated levels of heme and hemolytic diseases and angiogenesis. However, heme loss can change the morphology of mitochondria and their dynamics, causing an increase in ROS levels and harming the glycolytic capacity of ECs [241,242]. In the same vein, Vandekeere et al. have reported that heme depletion causes elevated ROS levels induced EC death. Moreover, mice deficient in phosphoglycerate dehydrogenase (*Phgdh*) can suffer vascular defects because of decreased EC proliferation and survival. However, heme supplementation in *Phgdh* knockout EC can restore ETC function and rescue defects in angiogenesis and apoptosis [244]. Furthermore, heme synthesis is essential for EC respiration, especially for Complex IV (COX IV) function, and its inhibition showed anti-angiogenic effects in retinal ECs in vitro and animal models of visual neovascularization [242]. Additionally, heme depletion reduces OXPHOS and mitochondrial COX IV in human retinal microvascular endothelial cells (HRECs) and murine retina ex vivo [242]. Further studies are required to fully understand the role of heme in angiogenesis associated pathophysiological conditions.

## 3. Diseases and Conditions Associated with Elevated Heme

### 3.1. Elevated Heme Levels Underly Lung Injury

Acute lung injury (ALI) is a significant risk factor after pulmonary resections for NSCLC [245]. ALI-caused acute respiratory distress syndrome (ARDS) may result in fulminant acute hypoxemic respiratory failure, bacterial infection, and death [246,247,248]. In a murine model of trauma hemorrhage (TH), heme triggers the TLR4 (toll-like receptor 4)-and HMGB1 (high mobility group box 1)-dependent mechanisms, increasing pulmonary edema, decreasing bacterial clearance, and further leading to lung bacterial infection after TH and stored red blood cells (RBCs) transfusion [249]. Interestingly, heme is also critically involved in toxic gas inhalation-induced ALI because of its unique gas-binding properties. Exposure of C57BL/6 mice to halogen gas (bromine (Br_2_), phosgene Carbonyl Chloride (COCl_2_), and chlorine (Cl_2_)) increases intravascular hemolysis, resulting in elevated heme levels in plasma and causing oxidative stress damage and inflammatory effects that lead to ARDS [250,251,252]. The treatment of animals with the heme-scavenging protein Hx attenuates heme levels in the lung and significantly decreases ALI induced by Br_2_ and Cl_2_ [251,252]. In vitro and in situ studies indicate that cell-free hemoglobin (CFH) mediated alveolar-capillary barrier disruption [3] and apically located amiloride-sensitive (ENaC) and cation sodium (Na+) channel damage [252] may be responsible for pathological events of post inhalation-induced ALI. Similar to ALI, patients with very severe chronic obstructive pulmonary disease (COPD), which is a significant factor for the increased incidence rate of lung cancer, have elevated plasma heme levels accompanied by the increased expression of endoplasmic reticulum (ER) stress marker Grp78/Bip [253]. Treating a mouse model of Br_2_-induced chronic lung injury with Hx also reduces plasma CFH and prevents evidence of chronic lung injury [253]. Overall, these studies show the association of heme and oxidative stress and highlight the important roles of elevated heme in trauma hemorrhage and inhalation-induced lung injury. Thus, scavenging heme can be a potential therapeutic approach in lung injury-related pathogenic events.

### 3.2. Elevated Heme Levels Affect Cardiac Physiology

Numerous studies have found an association between elevated levels of circulating heme and hemolytic diseases such as sickle cell disease (SCD), thalassemia, cardiac bypass, sepsis, and malaria. Moreover, intravascular cell and tissue damage have been linked to elevated extracellular heme levels due to the saturation of heme scavengers and heme degradation enzymes during severe hemolysis [254]. Heme scavengers Hx and Hp have anti-inflammatory properties in hemolytic diseases such as SCD and thalassemias, which can cause endothelial dysfunction and oxidative damage [255]. Exogenous administration of Hx prevents accumulation of heme–iron in the cardiovascular system and normalizes disease parameters such as high blood pressure and altered cardiac function in SCD Hx-null mice. Hence, Hx can work as a potential therapeutic drug against cardiovascular heme-induced dysfunction in hemolytic related disorders [255]. Hx and Hp levels in plasma are decreased in SCD mice and patients. However, induction of CO/HO-1 by these heme scavengers can inhibit hemoglobin and heme-mediated microvascular stasis in SCD hyper hemolytic mouse model, which suggests that hemoglobin-heme-dependent vaso-occlusive crisis (VOC) and chest syndrome in SCD patients can be prevented by Hp and Hx supplementation [256].

Similarly, another study found an association between heme from hemolysis and TLR4 signaling on inflammatory and ECs [257]. Labile heme acts as a damage-associated molecular pattern (DAMP) and binds cofactor soluble myeloid differentiation factor-2 (sDM2), activating endothelial TLR4 and causing activation of the endothelium and vaso-occlusion in a SCD mouse model [258]. Knockout of vascular TLR4 signaling reduced heme-dependent inflammation and VOC [257]. Thus, targeting Hx and Hp levels, vascular endothelial TLR4 inhibition, and reducing elevated levels of sMD2 can result in promising strategies in SCD treatment and other hemolytic conditions. Additionally, the 2015 review by Sawicki et al. summarized that the elevated circulating heme- and hemoglobin-induced ROS-dependent smooth muscle proliferation may further contribute to cardiovascular pathology [259]. Likewise, elevated levels of heme were found to be associated with elevated oxidative stress due to increased production of ROS and cell death in cardiac myoblasts [260].

The protective role of heme degradation enzyme HO-1 in vascular remodeling and atherogenesis has been a hot topic for the last decade [261]. However, the mechanisms underlying HO-1-based protection and the role of heme in HO-1 related cardiac pathology are not entirely understood. *HMOX1* plays an important role in the development of the placental vasculature and spiral artery remodeling, regulates vascular tone and inflammation, promotes endothelial growth and re-endothelialization after vascular injury, provides protection against EC apoptosis, and inhibits vascular smooth muscle cells (VSMC) growth [262]. Anti-inflammatory properties of HO-1 induction can slow the progression of atherosclerotic symptoms and regulate respiratory tissue homeostasis in cardiopulmonary heart disease, and increased levels of HO-1-dependent serum bilirubin improves prognosis in coronary artery disease and stroke patients [263]. Additionally, the CO/HO-1 system is crucial in mitochondrial biogenesis and cardiac development and differentiation of cardiomyocytes derived from spontaneous differentiated murine ESCs [264]. While there is no impact of HO-1 induction on cardiomyocyte differentiation and mitochondrial maturation in human-induced pluripotent stem cell-derived cardiomyocytes (hiPSC-CMs), possibly due to anatomical differences between both organisms, HO-1 knockout alters the electrophysiology of hiPSC-CMs [265]. Therefore, it is necessary to do further investigations to elucidate the effect of heme and HO-1 and their products on human cardiomyocytes maturation in physiological and pathological conditions.

### 3.3. Role of Heme as a Pro-Inflammatory Influencer and Hb-Derived DAMP

Hemolysis causes an increase of intravascular heme, oxidative damage, and inflammation in which macrophages play a critical role [266]. Once heme is released into the plasma, heme homeostasis is maintained by Hx and Hp. Excess heme can drain the body’s scavenging mechanism, resulting in increased labile heme in the system. Labile heme in uncontrollable amounts has deleterious effects. Labile heme can directly interact with serum components, influence host innate immune response, activate the complement system and the HO-1/ferritin system, maintain homeostasis by macrophages, and makes the host susceptible to bacterial infection due to increased iron level [266,267,268]. Thus, understanding the molecular mechanisms of regulating heme metabolism and the role of heme metabolites is crucial.

Labile heme has been recognized as a compelling pro-inflammatory conciliator. When released from hemoglobin, labile heme can become a DAMP [46]. Heme is well-depicted as Hb-derived DAMP that targets various immune and non-immune cells. Hb-derived DAMPs elevate ROS production, stimulate neutrophils, and increase proinflammatory cytokines [266]. Excessive heme directly causes oxidative stress and activates the unfolded protein response (UPR), causing renal injury [269]. The molecular mechanism of action of how heme is mediated in inflammation is under debate. Recent studies revealed that heme promotes ROS generation and activates spleen tyrosine kinase (Syk) activation and establishes memory by epigenetic changes [266]. These two features are critical for most proinflammatory signaling pathways [270]. The pro-inflammatory response is primarily mediated via TLR4 activation in macrophages [267]. Other studies have shown that heme provides a second signal for stimulating the processing of interleukin 1b (IL-1b) by the NLR family pyrin domain containing 3 (NLRP3) inflammasome and proinflammatory cytokine production in LPS-primed macrophages [266,268,271]. Heme can induce the formation of C3a and C5a, and the assembly of membrane-attack-complex (MAC), thus activating the complement system [266,272]. Anti-inflammatory responses can also be provoked by labile heme via the induction of the heme degradation enzyme [268,273]. Heme degradation product CO is a gasotransmitter with potent anti-inflammatory properties [274]. CO has been recently shown to completely elicit macrophage NLRP3 inflammasome activation in response to bacteria, subsequently inducing bacterial ATP production and following ATP sensing in macrophages [271].

The main function of the erythrocyte is to transport oxygen, which is carried out by hemoglobin containing heme with coordinated iron ion as the essential prosthetic group. The major source of iron is its recycling pathways. Macrophages scavenge obsolete and damaged erythrocytes to discharge iron from the hemoglobin and promote erythropoiesis [12]. Notably, iron regulation by macrophages becomes defective due to inflammation, which substantially affects iron homeostasis and erythropoiesis [275,276]. Macrophages and erythrocytes have a symbiotic relationship: erythrocyte derived heme can induce monocytes and neutrophil chemotaxis, and monocytes can recognize receptor pattern and activate iron-recycling macrophages [275].

It has been observed that people with hemolytic disorder are more susceptible to bacterial infections. A recent study shows that heme, independent of iron, can interfere with DOCK8-mediated Cdc42 activation and inhibit phagocytosis [273]. In addition, it can alter actin cytoskeleton remodeling and reduces host defense against bacterial infection [268]. Another study analyzed the effect of heme on neutrophils infected with *Leishmania infantum* causing visceral leishmaniasis [277,278]. Serum concentrations of heme are directly proportional to HO-1 and lactate dehydrogenase levels and inversely proportional to peripheral blood neutrophils counts [277]. Their experiments demonstrate infected neutrophils are stimulated by heme, promoting significant rises in superoxide dismutase-1 activity and HO-1 mRNA expression. Therefore, heme alone can elicit oxidative stress-related cell fatality. Hence, heme activates neutrophil function and oxidative stress which supports intracellular *L*. *infantum* endurance [277,278]. Therefore, one of the diverse roles of heme is mediated through anti-inflammatory and antioxidant effects. Complete understanding of the role of heme as DAMP and its associated inflammation could contribute to the advancement of novel therapeutics to deal with disease conditions.

### 3.4. Elevated Heme Metabolism Promotes Neurodegenerative Functions in the Nervous System but Is Perturbed in Alzheimer’s Dementia

Heme acts as a signaling molecule and is important for neurogenesis, neuronal growth, and survival. It facilitates neuroprotection by detoxifying neurotoxins resulting from oxidative stress via incorporation into neuroglobins. Therefore, participation of heme in several cellular pathways causes neuronal sensitivity to altered heme levels [279,280]. Changes in heme metabolism lead to changes in oxygen sensing and neuronal survival. During brain injury due to intracerebral or subarachnoid hemorrhages, excessive heme is released. This promotes oxidative damage, lipid peroxidation, apoptosis, and neuronal cell death [279,281,282]. Several studies indicate the association of heme for the maintenance of the peripheral nervous system (PNS) and altered heme metabolism causing neuropsychiatric disorders like Alzheimer’s disease (AD) and Parkinson’s disease (PD) [64,280,283].

Appropriate heme homeostasis is key for the proper functioning of central nervous system (CNS). Three primary regulatory systems control heme levels in mammals. First, Hp, considered a marker for blood–brain barrier (BBB) impairment, is responsible for the scavenging of hemoglobin [284]. High expression of this protein is linked to neurological diseases associated with disturbed BBB integrity, as detected in AD and PD patients [285]. Secondly, the plasma protein Hx prevents heme-mediated cytotoxicity by transferring excess heme from the circulation to HO [284,286]. Thirdly, HOs degrade these excess intracellular hemes releasing CO, Fe^2+^, and biliverdin [12,287]. The exact role of this enzyme in various stressful events is still not clear, and the biological effect of this enzyme can be tissue specific. It is believed that overexpressed HO-1 is impeccably receptive to stimuli provoking oxidative injury, providing neuroprotection [288]. Byproducts of heme degradation activate some signaling pathways enhancing expression of brain-derived neurotrophic factor (BDNF) in dopaminergic neurons and expression of glial cell derived neurotrophic factor (GDNF) in glia [289]. Besides, reduced heme metabolism contributes to lowered signaling intermediates like cAMP via the Ras-mitogen-activated protein kinase (MAPK) and its downstream target cyclic AMP-response element-binding protein, causing reduced neuronal differentiation [63,290].

In addition, heme transporters in the brain mediate intracellular and extracellular heme trafficking to prevent pathological outcomes resulting from disruption of homeostasis. Serious neuronal injury is observed in genetic inhibition of intracellular heme uptake transporters [286]. In comparison to other organs, the brain has elevated expression of HRG-1, which implies the significance of this transporter in maintaining brain heme homeostasis. The genetic reticence of FLVCR2, a heme importer of the FLVCR family, is associated with lethal autosomal disorder, which also lacks mitochondrial respiratory chain complex III and IV, implicating the importance of FLVCR2 in making heme accessible following incorporation into mitochondrial complexes [12,286]. Genetic mutations in extracellular heme traffickers mediated by ABCG2 and FLVCR expressed in brain cells have also been associated with impaired neurological functions. In the retina, ABCG2 averts oxidative damage and encourages the differentiation of neuronal stem cells. Aberrant expression of *FLVCR1* gene is also connected with compromised neuronal function causing degeneration of sensory neurons and development of Posterior column ataxia and retinitis pigmentosa (PCARP), thus indicating the involvement of heme in pain perception [12,291,292]. Hence, a series of compensatory mechanisms are engaged to inhibit the intracellular accumulation of heme. The salutary effect of maintaining heme hemostasis is the prevention of pathological outcomes associated with the disruption of heme regulation. 

Brain hemorrhages and intrusion of RBCs compromise the supply of oxygen and nutrients to neurons, causing discharge of heme, heme accumulation, and neurodegeneration, which leads to neurological disorders like AD [64,280]. Formation of Amyloid-β peptide (Aβ) senile plaques has long been associated with AD. Hemoglobin interacts with Aβ and co-localizes with Aβ plaques in AD post-mortem brains which exhibit peroxidase activity in the presence of H_2_O_2_ [293,294]. This link between hemoglobin expression and AD pathogenesis is corroborated with increased hemoglobin levels observed in amyloid pathology correlated brain areas—cerebral parietal gray matter, inferior temporal gyrus, and parietal white matter [295]. Recent evidence also suggests heme homeostasis is perturbed in AD [293,296,297]. Analysis of gene expression of AD vs. normal brain tissues identified heme related gene *ALAS1*, a rate-limiting enzyme in heme synthesis, and HO-2, whose expression is lowered in hippocampi of AD brains and APPPS1 mouse brains, suggesting its importance in AD hippocampi [296,298]. Hence, lowered heme metabolism is suggested to be an early onset sign of AD pathogenesis [296,299]. More understanding of the dynamic range of heme foraging in the brain will offer a precious tool to resolve the involvement of heme-mediated cytotoxicity in promoting neurodegenerative diseases.

### 3.5. Elevated Heme Is Associated with Impaired Glucose Tolerance and Insulin Resistance in Type II Diabetes Mellitus while Intracellular Heme Deficiency Attenuates Mitochondrial Activity and Impairs Glucose Metabolism

Type II Diabetes Mellitus (T2D), the most common endocrine disorder, is a chronic metabolic disease characterized by insulin resistance and eventual inability of the pancreas to secrete insulin, resulting in hyperglycemia that over time damages body tissues such as nerves and blood vessels [300]. In Western countries, dietary heme makes up two-thirds of the body’s iron reservoir, even though it constitutes only one-third of ingested iron [1,301]. In fact, a positive correlation was found to exist between heme iron intake and risk of T2D in several epidemiological studies [302,303,304,305,306,307,308,309]. Additionally, a positive correlation exists between cancer and T2D, with diabetics being at an increased risk of colon cancer, breast cancer, pancreatic cancer, liver cancer, endometrial cancer, bladder cancer, and non-Hodgkin’s lymphoma [310,311,312,313,314,315,316]. Furthermore, T2D increases risk of cancer mortality when controlled for other factors [317,318].

Altered systemic glucose metabolism, indicative of T2D and metabolic syndrome, is associated with increased heme tissue levels and export, as seen by the increased expression of the plasma heme exporter FLVCR1 in adipose tissue of patients with T2D [319]. FLVCR1 mRNA is positively correlated with fasting glucose, fasting triglycerides, serum ferritin, blood hemoglobin, hematocrit, and % change in fasting glucose in an independent cohort, as well as negatively correlated with insulin sensitivity [319]. Beta Thalassemia Major patients and pediatric bone marrow survivors, both of which receive a high number of blood transfusions leading to high plasma heme levels as a result of erythrocyte lysis, have an increased risk of T2D [320,321,322,323,324]. Additionally, impaired heme clearance plays a role in T2D [325,326,327] and upregulation of heme clearance pathways yields therapeutic benefit in diabetic myocardial infarction [328]. Interestingly, metformin, the most commonly used drug to treat T2D [329,330], was shown in a 2018 study by Li et al. to suppress heme production by 50% in yeast and 30–50% in human erythrocytes, erythropoietic cells, and hepatocytes, and to prevent heme oxidation in cytochrome C, myoglobin, and hemoglobin [329]. The above studies agree with epidemiological studies and point towards elevated plasma and tissue heme levels as being associated with T2D, as well as pointing towards targeting heme as a potential therapeutic strategy.

While the mechanism of action of heme in T2D is not clear, heme can directly act on proteins involved in glucose regulation. Heme is demonstrated to bind to insulin using two-heme binding sites (Kd = 3.13 µM), enhancing its peroxidase activity [331]. The heme–insulin complex leads to insulin cross-linking, effectively causing loss of insulin function and enhancing protein tyrosine nitration, which leads to inactivation of proteins involved in T2D [331]. Additionally, islet amyloid polypeptide (IAPP), whose deposition within the β-cells of the islets of Langerhans is implicated in β-cells death and diabetogenesis, is able to bind heme [332]. Heme–IAPP can produce partially reduced oxygen species, inducing oxidative stress in β-cells [332]. The above evidence suggests that dietary heme intake, as well as elevated plasma and tissue heme, are associated with hyperglycemia and insulin resistance and may directly affect key proteins implicated in T2D, leading to diabetogenesis.

Heme is incorporated into several mitochondrial complexes and is necessary for proper mitochondrial functioning [1]. While elevated heme levels are observed in diabetics and may lead to insulin resistance and other diabetic hallmarks, conversely, heme deficiency may lead to attenuated mitochondrial activity associated with T2D. This may be due to the difference between total cellular heme versus the “regulatory heme pool”, as described by Saitoh et al. [333]. In mice heterozygous null for ALAS1, a key heme synthesis enzyme, they observed impaired glucose tolerance and insulin resistance after 20 weeks. However, in murine skeletal muscle tissue they could not observe any significant reduced heme content, even after several quantifications, although ALAS1 mRNA levels were halved due to heterozygous knockout. Improvement in impaired glucose tolerance and insulin resistance was observed after treatment with 5-aminolevulinic acid (ALA), a heme precursor, after only one week, indicating effects observed were due to decreased heme in the “regulatory heme pool”. Studies in myocytes confirmed ALAS1 knockdown reduced insulin-stimulated glucose uptake response and treatment with ALA led to recovery, demonstrating that the role of ALA deficiency occurs in a cell-autonomous manner. Succinylacetone, an inhibitor of 5-aminolevulinate dehydratase needed for the subsequent step in the heme synthesis pathway after ALA, decreased insulin-stimulated glucose uptake response, indicating heme deficiency in the “regulatory heme pool” is responsible for the impaired glucose tolerance seen in ALA deficiency [333].

Inducible hepatic porphyrias, which are inherited disorders in heme biosynthesis that lead to toxic buildup of heme-intermediates, can be treated with high glucose load, which is thought to decrease ALAS1 expression, further contributing to the relationship between heme and glucose metabolism [333,334]. Additionally, supplements of ALA in cohort studies show therapeutic benefit in mildly hyperglycemic and prediabetic patients [335,336]. These results indicate that in addition to elevated heme levels in tissue and plasma, which may affect key protein activity leading to diabetogenesis, low heme levels in the “regulatory heme pool” of the cell may attenuate mitochondrial activity and disrupt glucose metabolism, leading to a dual role of heme in T2D.

## 4. Conclusions

**Figure 3:**
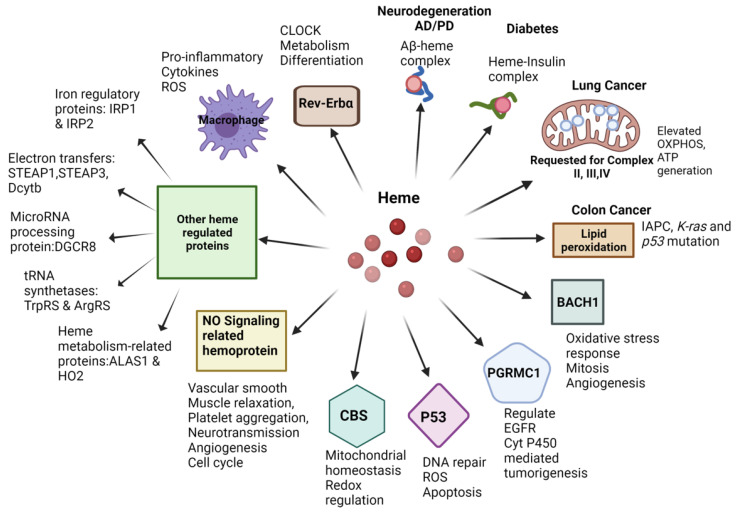
Illustration summary of heme regulation in diverse molecular and cellular processes. (Created with BioRender.com).

This review summarizes recent literature on the association of heme and fundamental processes involved in the development of cancers and other related diseases (Figure 3). Elevated heme metabolism is notably found to sustain OXPHOS and promote proliferation and tumorigenesis of tumors like NSCLCs. In addition, alterations in heme metabolism are directly involved in promoting pancreatic and CRC, while dietary heme intake may play a role in CRC. Heme degradation carried out by HO-1 is also fundamentally involved in the pathologies of diverse cancer types. Moreover, heme acts as a regulator that modulates various cellular processes by binding crucial transcription regulators and cancer-related proteins such as BACH1, PGRMC1, P53, CBS, sGC, and NOS. Heme dysregulation causes severe consequences in angiogenesis, immune response, neurogenesis, and circadian rhythm, all potentially contributing to the related tumor development. Recent studies show that targeting heme and heme mechanisms is likely to be a new therapeutic strategy in many diseases including cancer treatment. For example, Hx scavenges labile heme and normalizes heme-induced dysfunction in SCD [255] and acute and chronic lung injury [251,253]. Mitochondrially targeted deferoxamine (mitoDFO) that chelates mitochondrial [Fe-S] clusters/heme iron suppresses proliferation and migration and induces cell death in varied cancer types, including breast, ovarian, and pancreatic cancers [24]. Inhibition of heme uptake and heme synthesis by heme-sequestering peptides (HSPs) and cyclopamine tartrate (CycT), respectively, represses lung tumor growth [21,26,28]. Altered heme levels and heme metabolism are also implicated in other diseases, including hemolytic disorders, neurodegenerative diseases such as Alzheimer’s dementia, and diabetes mellitus. Hence, the characterization of heme-associated pathogenesis and regulators will advance the study of potential therapeutic approaches targeting heme for the treatment of cancer and other diseases.

## Figures and Tables

**Table 1:** Heme regulated multifunctional signal transducers and regulators involved in disease diagnoses and treatments.

Protein Name	Binding Residues	Binding Affinity	Heme Function	Heme in Proteins Involved in Pathogenesis of Disease	Reference
BACH1	CP motifs in HRM3-6	Kd = 1.37 × 10^−5^ M (C-terminal region)	Promote the dissociation of BACH1 from DNA; promote nuclear export and ubiquitination of BACH1	Heme destabilizes BACH1, leading to the inhibition of different types of cancer	[136,143,144,145]
PGRMC1	Tyr113, Tyr107, Lys163, and Tyr164	Kd = 50 nM	Mediate PGRMC1 dimerization	Mediates PGRMC1 regulated EGFR and cytochromes P450 activity in colon cancer cells and hepatoma cells	[138,146,147]
P53	Cys275/Cys277	Kd = ~1.2 µM	Mediate P53 destabilization	Heme–P53 may mediate colon carcinoma cell suppression based on iron-deprivation	[139]
CBS	Cys52/His65 Cys15/His22	Kd = 2.18 ± 0.64 µM (Cys15/His22)	Promote CBS folding and assembling	Lack of in vivo study. Limiting heme inhibits CBS activity without abolishing the enzymatic activity in vitro.	[140,148,149]
sGC	His105 in β1 subunit	Contains heme as a cofactor	Cause conformational change to initiate the first step in sGC activation	A cofactor that is required for essential sGC activity	[150,151,152]
NOS	NOS1/nNOS: Cys419 NOS2/iNOS: Cys184 NOS3/eNOS: Cys200	Contains heme as a cofactor	Cofactor to NOS which catalyzes NO synthesis	Heme is required to maintain basic enzyme function including NOS homodimerization and catalysis of NO synthesis	[153,154,155]
